# Clubfeet and congenital constriction band syndrome

**DOI:** 10.1186/s40001-021-00492-z

**Published:** 2021-02-16

**Authors:** Bujar Shabani, Dafina Bytyqi, Cen Bytyqi

**Affiliations:** 1 Department of Orthopaedics, University Clinical Center of Kosova, Pristina, Kosovo; 2 Faculty of Medicine, University of Prishtina, Pristina, Kosovo

**Keywords:** Clubfeet, Ponseti procedure, Constriction band

## Abstract

**Background:**

Clubfeet and constriction band syndrome is a very rare non-idiopathic condition. Treatment is often difficult and the recurrence deformity rate is high. The purpose of this study was to assess the effectiveness of Ponseti method in the treatment of congenital constriction band syndrome accompanied by clubfoot deformity and lymphedema.

**Case presentation:**

We are presenting an interesting case of bilateral clubfeet and congenital circumferential constriction band syndrome in the lower limb. Ponseti method of correcting the congenital clubfoot deformity was applied. Constriction band release is accomplished by two stages completely excising the fibrous band and multiple two-stage Z-plasties on the right calf.

**Conclusion:**

The results of this study indicate that the Ponseti method of gentle, systematic manipulation and weekly cast changes is an effective treatment of non-idiopathic clubfoot distal to congenital amniotic constriction band.

## Introduction

The constriction amniotic band syndrome (CABS) is a collection of fetal malformations associated with fibrous bands that appear to entangle various fetal parts in utero, leading to deformation malformation, or disruption [1, 2]. The amniotic band syndrome occurs sporadically and the incidence is one case per around 15,000 live births. It is characterized by compression in the soft tissue usually involving the deep fascia surrounding the leg at the time of birth [3, 4]. The fact that many theories attempt to explain the etiology of amniotic band syndrome suggests that the etiology of this condition remains unknown [1]. An associated clubfoot deformity has been reported to occur in between 12 and 56% of patients with CABS [4, 5]. The involved foot may occur below an ipsilateral band or appear in a limb without a proximal band. Some authors have found the clubfeet that are associated with this syndrome to be rigid and resistant to nonoperative management, and the majority of patients in a number of early series were treated by extensive surgical release [5–7] Clubfoot combined with circumferential amniotic band syndrome is distinguished from other types of congenital deformity because of risk of multiply relapses [6, 8, 9]. According to Ponseti under ultrasound examination, a clubfoot is rarely detected before the 16th week of gestation [10]. When a clubfoot deformity is suspected during prenatal ultrasound screening this should lead to a more thorough search for co-morbidity [11]. This case is reported because of its extreme rarity of bilateral congenital constriction band syndrome accompanied by clubfoot deformity and lymphedema. The purpose of the present study was to evaluate the results of the Ponseti method for the treatment of clubfoot associated with constriction band syndrome.

### Case presentation

**Fig. 1:**
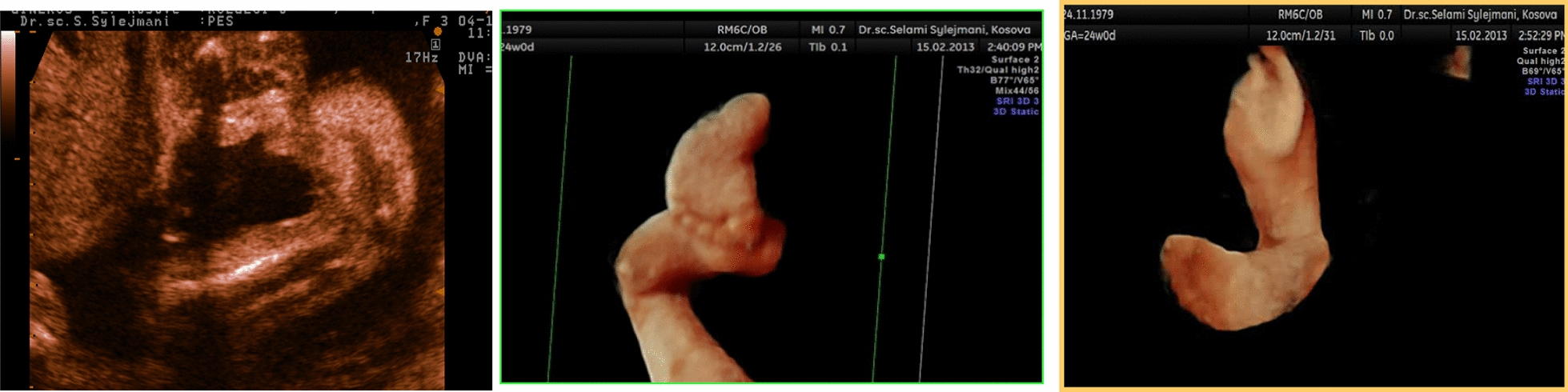
Ultrasound two- and three-dimensional images of clubfoot at 18 weeks of gestation

A male child of Caucasian origin was brought to our hospital at the age of 5 days with circumferential congenital constriction rings just above the ankle joint, and severe clubfeet. The pediatric examination revealed no other abnormality in the upper extremities or other organs. Pregnancy was uneventful, but during a routine ultrasound examination at 18 weeks of gestation, the gynecologist noticed bilateral clubfoot deformities and informed the parents (Fig. 1). The child was born by normal vaginal delivery at full term with cephalic presentation. There was no family history of congenital anomalies. The karyotype test was done and revealed no abnormality. Further genetic testing was not done.

**Fig. 2:**
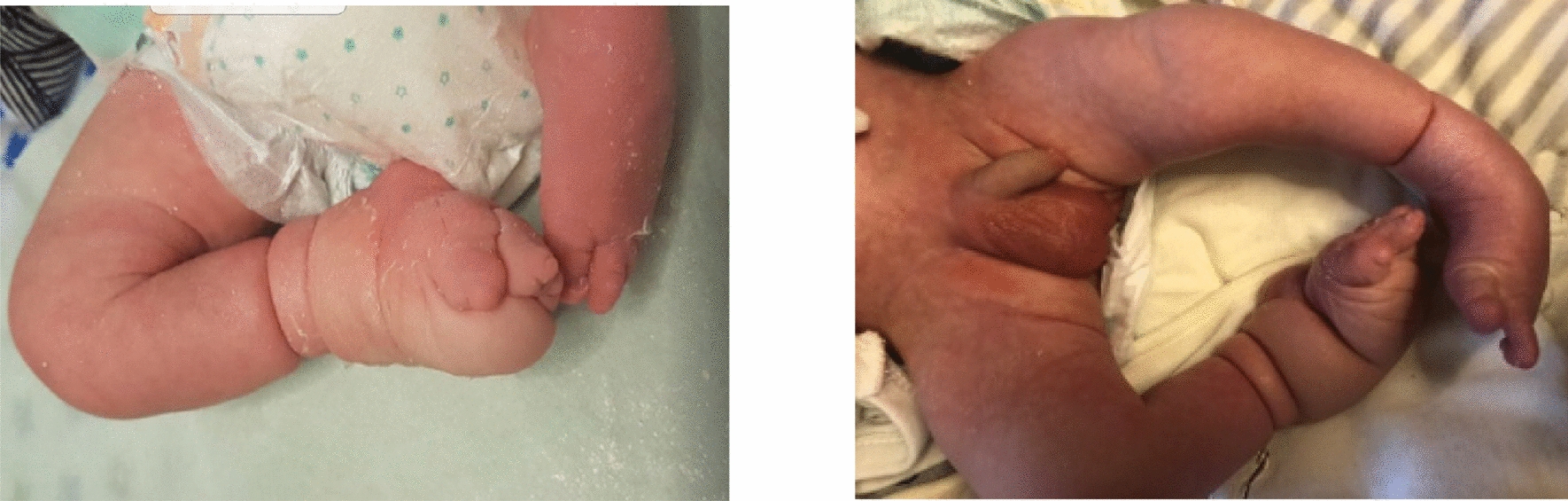
Severe bilateral clubfoot in a newborn at the day of birth and at the age of 5 days with constriction rings. Constriction rings in the calf above to the ankle joint are circumferential and responsible for foot and ankle lymphedema on the right side

On inspection, both feet had inversion at the subtalar joint, equinus and varus in the ankle joint, adduction of the forefoot, pronation of the forefoot in relation to the ankle joint, cavus (excavatum), internal rotation of the crural region. (Fig. 2) The right foot: a constriction circumferential ring, type II in Patterson classification, was located about 4.0 cm above ankle joint without neurologic deficit, but with dorsal lymphedema. The toes of the right foot were hypoplastic. There was also a severe clubfoot deformity grade 4 according to Dimeglio classification with a marked medio-tarsal crease. The left foot: a circumferential constriction band, type II in Patterson classification, was located above 3.8 cm on the ankle but without lymphedema. The clubfoot deformity was grade 4 according to Dimeglio. There was not noticed any limb-length discrepancy.

Clubfeet were corrected with Ponseti method as a safe and effective procedure. During treatment of clubfoot, three phases were essential: reduction of deformation (2 months), consolidation of obtained results (4 months), and manage the risk for relapse (3 months). A long-leg-cast was applied with knee flexed 90°. The cast was changed every week with gradual correction of the deformity according to Ponseti protocol. Cavus, adductus, and varus were fully corrected but dorsiflexion was limited for 20 degrees bilaterally, so the tenotomy of the Achilles tendon was indicated. Because of lymphedema from amniotic band syndrome on the right foot, we decided to cut the constriction bands to release the tourniquet-like effect. Percutaneous Achilles tenotomy was performed bilaterally and three longitudinal incisions were made through the constriction band, on the right foot.

**Fig. 3:**
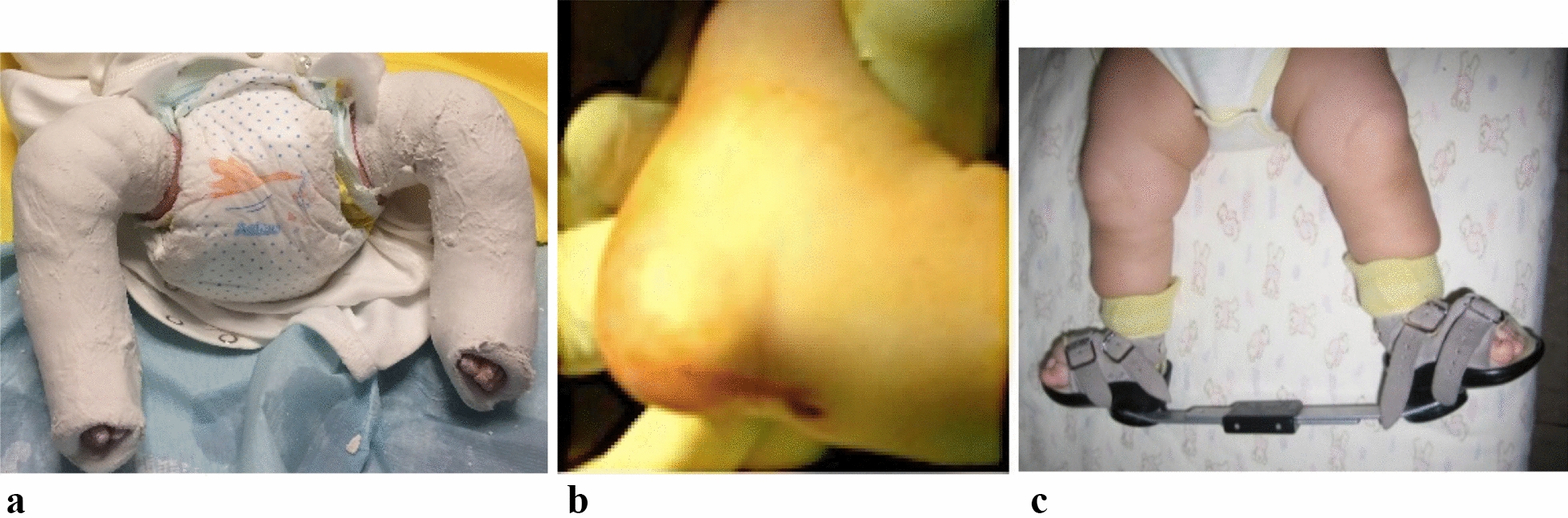
Clubfeet were corrected with Ponseti method using plaster of Paris in our case because of the possibility of more precisely moulding in comparison to fiberglass (**a**). Because full dorsiflexion was not possible with stretching alone, a percutaneous Achilles tenotomy was performed (**b**). The foot abduction brace (FAB) was crucial to maintain correction and preventing relapse (**c**)

**Fig. 4:**
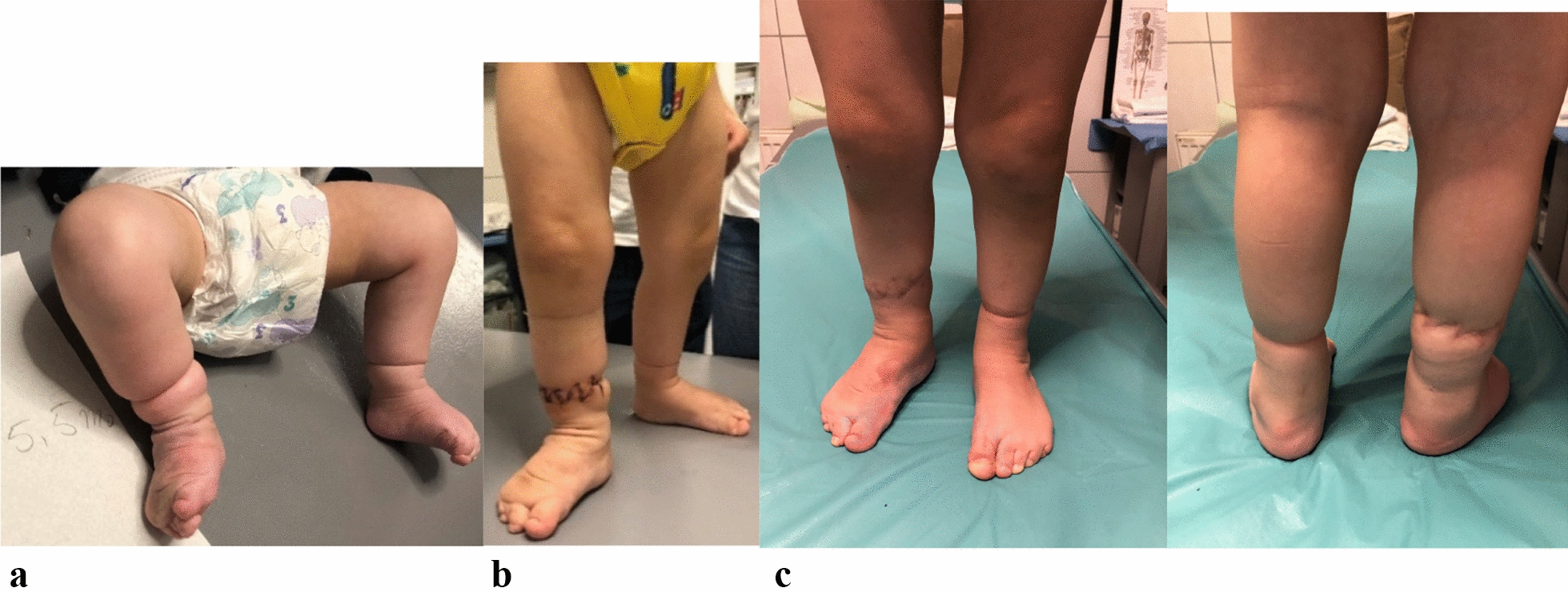
Preoperative view at age of 5.5 months after Ponseti procedure. Deformation and lymphedema improved (**a**). Postoperative view after excision and repair in two stages of constriction band. Complete dissection in two stages of the fibrous band improves significantly lymphedema (**b**). The last follow-up at age of 2 years, there is no sign of relapse and the lymphedema is recovered (**c**)

Of course, a later reconstruction procedure was necessary. There were no bleeding complications following percutaneous Achilles tenotomy (Fig. 3b). The post-tenotomy casting remained for 3 weeks with changing the cast every 10 days. After Ponseti procedure, the foot abduction brace (FAB) protocol was applied to maintain the correction: the bracing protocol included 23 h a day at 700 of external rotation for 3 months, then reduced to 18 h a day and then removed gradually, 1 h a day until use of 12 h a day. After walking age, the brace was worn at night (Fig. 3c). The patient was followed until 2 years old, and there was no relapse of the deformity. A two-stage circumference excising of the congenital constriction band was done by plastic surgeons, with a 3 months interval between stages (Fig. 4). The first stage of surgery was done at age of 13 months and 3 weeks, and the second stage 3 months later. There was no wound complication. The lymphedema was withdrawn.

## Discussion

In our case, clubfeet associated with constriction bands were successfully treated with Ponseti method and multiple Z-plasties. Tada et al. reviewed a series of 83 patients with congenital annular constricting bands and found 19 children with clubfeet [4]. Askins and Ger reported a 49% association of congenital band with clubfeet [12]. Most of the clubfeet associated with CABS that have been reported in the literature to date have been managed by extensive soft tissue release. Auington et al. assessed the outcome of treatment in 18 patients with clubfeet distal to a congenital band, the majority of which were treated using extensive surgical release [13]. Syndromic clubfeet have been reported to be resistant to nonoperative management, and complete releases are routinely required. In patients with constriction band syndrome, Hennigan and Kuo [6] noted 62% good results. The results of surgery have often been inconsistent. Recently, because of the good outcomes with idiopathic clubfeet, the Ponseti method has been used for non-idiopathic cases, also. Advantages of the Ponseti method are: high-quality reduction of clubfoot with the restoration of a sub-normal anatomy, low cost, and small displeasing worry for the parents. Fortunately, the Ponseti strategy in our case worked well and no relapse. Carpiaux et al. reported that ipsilateral banding did not affect the effectiveness of the Ponseti technique in the treatment of clubfeet associated with CABS [14]. Zionts et al. also reported that all the clubfeet in their series were corrected by a series of manipulation and cast applications using the Ponseti technique [8]. But, Jackson et al. concluded that clubfoot associated with constriction band required more casts to achieve an acceptable correction and had an increased risk of deformity recurrence compared with subjects with isolated clubfoot [15]. The constriction circumferential band on the right calf in our case was operated by plastic surgeons in two stages, during the first stage, anterior part of the constriction band was released and in the second stage, posterior part. A higher number of cases and a longer follow-up period is needed to evaluate the long-term efficacy of Ponseti method in clubfoot associated with constriction band syndrome. Another limitation of our study is that because of lack of facilities, it was not possible to do thorough genetic tests, other than karyotyping.

## Conclusion

Clubfeet associated with constriction rings syndrome are challenging deformities. Relapse rates (recurrence of a previously corrected deformity) are high and should be taken into consideration. The results of this study indicate that the Ponseti method of casting is an effective treatment of clubfoot in newborns with constriction rings syndrome.

## Data Availability

All data generated or analysed during this study are included in this published article.

## References

[1] Ross MG (2007). Pathogenesis of amniotic band syndrome. Am J Obstetrics Gynecol..

[2] Pais M, Lewis LE, Bhat RY, Jayashree P, Pais M (2016). Amniotic band sequence with clubfoot Amniotic band sequence with clubfoot in a neonate: a case report. Manipal J Nursing Health Sci..

[3] Patterson T (1969). Ring constrictions. Hand.

[4] Tada K, Yonenobu K, Swanson AB (1984). Congenital constriction band syndrome. J Pediatr Orthop.

[5] Gomez VR (1996). Clubfeet in congenital annular constricting bands. Clin Orthop Relat Res.

[6] Hennigan SP, Kuo KN (2000). Resistant talipes equinovarus associated with congenital constriction band syndrome. J Pediatr Orthop.

[7] Synder M, Niedzielski K, Grzegorzewski A (2000). Surgical treatment of congenital clubfoot with constriction band syndrome. Chir Narzadow Ruchu Ortop Pol.

[8] Zionts LE, Habell B (2013). The use of the ponseti method to treat clubfeet associated with congenital annular band syndrome. J Pediatr Orthop.

[9] Chang CH, Huang SC (1998). Clubfoot deformity in congenital constriction band syndrome: manifestations and treatment. J Formos Med Assoc.

[10] Ponseti IV, Zhivkov M, Davis N, Sinclair M, Dobbs MB, Morcuende JA (2006). Treatment of the complex idiopathic clubfoot. Clin Orthop Relat Res..

[11] Sylejmani S, Syla B, Shala S. Diagnosis of congenital anomalies during routine fetal surveillance. Donald School J Ultrasound Obstetrics Gynecol. 2015.

[12] Askins G, Ger E (1988). Congenital constriction band syndrome. J Pediatr Orthop..

[13] Auington NJ, Kumar SJ, Guille JT (1995). Clubfeet associated with congenital constriction bands of the ipsilateral lower extremity. J Pediatr Orthop..

[14] Carpiaux AM, Hosseinzadeh P, Muchow RD, Iwinski HJ, Walker JL, Milbrandt TA (2016). The effectiveness of the Ponseti Method for treating clubfoot associated with amniotic band syndrome. J Pediatr Orthop.

[15] Jackson T, Jones A, Miller N, Georgopoulos G (2019). Clubfoot and tethered cord syndrome: results of treatment with the Ponseti method. J Pediatr Orthop.

